# Did the National Lockdown in Saudi Arabia Reduce Lower Respiratory Illnesses in Children?

**DOI:** 10.3389/fped.2021.717739

**Published:** 2021-11-18

**Authors:** Nasser S. Alharbi, Yossef Alnasser, Ahmed S. Alenizi, Alnashmi S. Alanazi, Abeer H. Alharbi, Faisal O. AlQurashi, Ibrahim Nafisah, Abdullah A. Yousef

**Affiliations:** ^1^Department of Pediatrics, College of Medicine, King Saud University, Riyadh, Saudi Arabia; ^2^Department of Pediatrics, King Saud University Medical City, Riyadh, Saudi Arabia; ^3^King Saud Medical City, Riyadh, Saudi Arabia; ^4^Security Forces Hospital, Riyadh, Saudi Arabia; ^5^Department of Pediatrics, Imam Abdulrahman Bin Faisal University, Dammam, Saudi Arabia; ^6^King Fahd University Hospital, Imam Abdulrahman Bin Faisal University, Khobar, Saudi Arabia

**Keywords:** lockdown, COVID-19, asthma, pneumonia, bronchiolitis, children

## Abstract

**Objectives:** This study aims to explore the effect of lockdown and early precautionary measures implemented in Saudi Arabia on number of pediatric hospitalizations due to lower respiratory illnesses (bronchiolitis, asthma, and pneumonia).

**Methods:** This is a retrospective cross-sectional study aims to review patients from four major hospitals in Saudi Arabia. All pediatric hospitalizations secondary to asthma, bronchiolitis, and pneumonia during the months of the lockdown (March, April, and May) in 2020 were documented. Then, they were compared to the previous 2 years. Variables like number of hospitalizations, oxygen requirement, mechanical ventilation, admission to the intensive care unit (ICU), length of stay, and results of viral studies were collected.

**Results:** We included 1,003 children from four different centers. Males were slightly higher than females (55.8% vs. 44.2%). Total number of hospitalizations in 2020 was 201, significantly lower than 399 and 403 hospitalizations in 2019 and 2018, respectively (*P* < 0.01). The major drop happened on the months of April and May. Although bronchiolitis hospitalizations' dropped by more than half in 2020 compared to the previous 2 years, it was not statistically significant (*P* = 0.07). But, asthma hospitalizations were significantly less in 2020 compared to the previous 2 years (49–65% reduction, *P* = 0.003). Number of pneumonia cases were lowered in 2020 compared to the previous 2 years. However, proportion of pneumonia diagnosis to total hospitalizations increased in 2020 (55% compared to 50% and 35%). There was a surge of viral testing during a period of uncertainty in the early phase of the pandemic. This total reduction in hospitalization was not associated with higher oxygen requirements, mechanical ventilation, ICU admissions or longer hospital stay.

**Conclusions:** Lockdown and precautionary measures executed during the early phase of COVID-19 pandemic helped decrease the number of hospitalizations due to lower respiratory illnesses in Saudi Arabia. Reduction in hospitalizations seems less likely to be secondary to hospital avoidance or delayed presentations as number of ICU admission and oxygen requirements did not increase. The post pandemic pattern of respiratory illnesses among children needs further research.

## Introduction

In December 2019, an outbreak was caused by a novel enveloped ribonucleic acid coronavirus in Wuhan, Hubei province, China (1). The coronavirus disease (COVID-19) outbreak was declared a pandemic by the World Health Organization (WHO) on March 12, 2020 (2). The WHO released an international technical guidance protocol for COVID-19 prevention and confinement during the pandemic (3).

In Saudi Arabia, the first case of COVID-19 was reported in a citizen on March 2, 2020 (4). Following this, the Saudi government gradually implemented precautionary health actions. Such precautions included a curfew (started on March 21, 2020) and cancellation of religious, social, and business gatherings (5). At the same time, awareness campaigns educating the public about basic personal protective measures were broadcast on all media outlets (6).

Viral respiratory illnesses (VRI) among children account for most hospitalizations (7). They can lead to a number of different respiratory diseases, including bronchial asthma, bronchiolitis, and pneumonia (7, 8). Although the focus of strict public health measures during the pandemic was to minimize the transmission of COVID-19, the time of the pandemic offers a unique opportunity to study the effectiveness of these measures on other viral-triggered illnesses. This study aimed to determine whether viral infection-triggered pediatric hospitalizations for asthma, bronchiolitis, and community-acquired pneumonia were affected by the lockdown and other measures to control COVID-19. Our main hypothesis that implementing a lockdown and strict public infection control measures to limit the transmission of COVID-19 would be reflected in the transmission of other respiratory viruses and their illnesses.

## Methods

### Study Design and Participants

The study employed a retrospective cross-sectional design. It involved reviewing charts from four tertiary centers in Saudi Arabia: King Khaled University Hospital (Riyadh), King Saud Medical City (Riyadh), Security Forces Hospital (Riyadh), and Imam Abdulrahman bin Faisal University Hospital (Dammam). After the inclusion and exclusion criteria were set, the study enrolled all hospitalized patients aged 14 years and younger with one of the following diagnoses: bronchiolitis, asthma, or pneumonia. The choice of 14 years of age reflects the pediatric inpatient admission criteria in Saudi Arabia. The study excluded children with confirmed COVID-19 or other diagnoses. The study compared the 3-month period of March, April, and May for each year from 2018 to 2020.

### Data Collection

All of the pediatric patients who were hospitalized during the study period were screened. Electronic charts of the patients who met the inclusion criteria were reviewed. Demographic, clinical, and laboratory data were also recorded. These included age, sex, clinical diagnosis, requirement for non-invasive oxygen supplementation, intensive care unit (ICU) admission, need for ventilatory support, viral study results, and length of hospital stay. All of these data were obtained and saved on encrypted Excel sheets (Microsoft Corporation, Albuquerque, NM, USA). Each day, 5–10 charts were reviewed. Subsequently, the data were entered into the Excel program. Any missing data in the electronic records was supplemented by a manual review of hard copy charts.

### Statistical Data Analysis

Mean and standard deviation were used to describe continuous variables, frequencies, and percentages. Multiple response dichotomy analysis was used to describe the children's admitting diagnoses. The chi-squared (χ^2^) test was used to compare differences between admissions over the years. Multivariate binary logistic regression analysis was used to understand the links between the children's diagnoses and their sociodemographic characteristics, time-related factors, and clinical presentations. The univariate non-parametric chi-squared (χ^2^) test of goodness-of-fit was used to assess the distribution of the lower respiratory infection admissions across the years for statistically significant differences. Enter methods were used to generate regression models. The associations between the patients' risk factors and their odds of admission with these diagnoses were expressed as a multivariate adjusted odds ratio (OR) with an associated 95% confidence interval (CI). The commercially available SPSS IBM V21 statistical analysis program (IBM Corp., Armonk, NY, USA) was used for data analysis, and the alpha significance level was set at 0.05.

### Study Ethics

The study was reviewed and approved by King Saud University's research ethical committee with study reference number 20/0504/IRB. All of the charts were retrospectively reviewed between June 2020 and June 2021. The authors declare no conflicts of interest.

## Results

### Study Enrollment and Demographics

A total of 1,003 pediatric patients were recruited from the four different centers. All of the patients were discharged at the time of data collection. The majority of the patients were infants and toddlers. The mean age at admission was 2.8 years (SD 3.034). The lowest number of admissions was for early adolescents (3.6%). Sex was almost equally distributed, with males being slightly higher (55.8% males and 44.2% females; Table 1).

**Table 1:** Demographic data of enrolled patients from four different centers from 2018 to 2020 in the months of March, April, and May.

	**Number**	**Percentage**
**Sex**		
Female	443	44.2%
Male	560	55.8%
Age (years), mean		2.85 (3.034)
**Age group**		
Infant ≤ 1 years old	409	40.8%
Toddler 2–3 years old	330	32.9%
Pre-schooler 3–7 years old	156	15.6%
School age 8–10 years old	72	7.2%
Adolescent ≥11 years old	36	3.6%
**Admitting hospital**		
King Saud Medical City	295	29.4%
King Fahad Medical City	150	15%
King Khalid University Hospital	272	27.1%
Special Forces Hospital	286	28.5%

### Number of Hospitalizations

The total number of hospitalizations for lower respiratory illnesses in March, April, and May 2020 was 201, in comparison to 403 and 399 hospitalizations in the same months in 2018 and 2019, respectively. The smaller number of hospitalizations in 2020 was statistically significant (*P* < 0.01). No statistical difference was found in the hospitalization count in 2019 compared to that of 2018 (*P* = 0.47). Subgroup analysis showed that this significant finding was driven by a significant drop in the rate of hospitalizations in April and May 2020 (the lockdown months) compared to the same months of 2018 and 2019. In March 2020, the rate of hospitalization was slightly lower than in the previous 2 years, but the difference was not statistically significant (Table 2).

**Table 2:** Number of hospitalization per year and per month.

	**2018**	**2019**	**2020**
Year of admission	403	399	201
Admission month		
March	212	196	176
April	120	119	18
May	71	84	7

### Diagnoses

A subgroup analysis of the number of hospitalizations based on diagnosis (bronchiolitis, asthma, and pneumonia) showed a reduction in the number of cases of bronchiolitis by more than half of the previous 2 years (55–62%). Despite being statistically insignificant (*P* = 0.07), this might be significant in terms of clinical situations and public health. This reduction paralleled the number of admitted asthma cases. However, it was statistically significant for asthma. The number of pneumonia cases in 2020 was lower than that in the previous 2 years. However, the proportion of pneumonia cases to total admissions was higher in 2020. Multivariate regression analysis illustrated a higher probability of being admitted to the hospital if you had a diagnosis of pneumonia in 2020 (OR 2.07; Table 3).

**Table 3:** Number of hospitalizations per year for each diagnosis.

**Diagnosis**	**2018**	**2019**	**2020**	**2020 Proportion**	***P*-value**
Bronchiolitis	165	142	64	31.80%	0.070
Asthmatic exacerbation	127 (31.5)	87	45	22.40%	0.003
Pneumonia	141 (35)	201	111	55.20%	<0.001

### Oxygen Requirement

**Figure 1:**
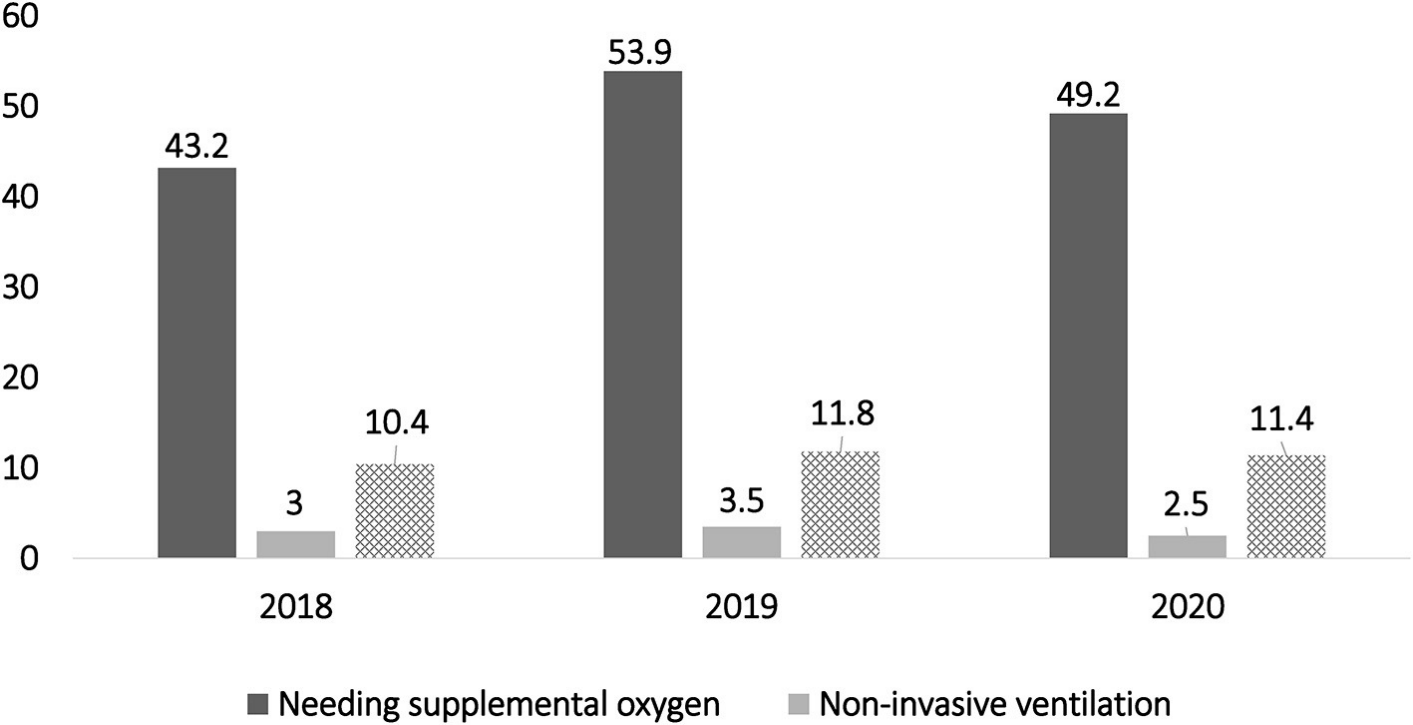
Indicates similar patterns of oxygen and ventilation requirements during the early phase of the coronavirus disease pandemic compared to the previous 2 years.

The percentage of patients needing oxygen supplementation during the study months in 2020 was 52.2% (*n* = 105). This was not statistically different from the findings during the same period in the previous 2 years (46.2% and 57.4% in 2018 and 2019, respectively). Similarly, the number of patients requiring non-invasive ventilation support was almost equal (Figure 1).

### ICU Admission and Ventilation Support

Children needing an ICU stay in 2020 represented 11.9% of the total hospitalizations (*n* = 24). However, this was not statistically different from the outcomes during the same period in the previous 2 years (16.1% and 13.3% in 2018 and 2019, respectively; *P* = 0.3). The percentage of patients who needed ventilation support during hospitalization in 2020 was 11.9% (24 patients). This was not statistically different from the results during the same periodin the previous 2 years (10.4% and 12% in 2018 and 2019, respectively; *P* = 0.4).

### Viral Testing and Length of Stay

**Figure 2:**
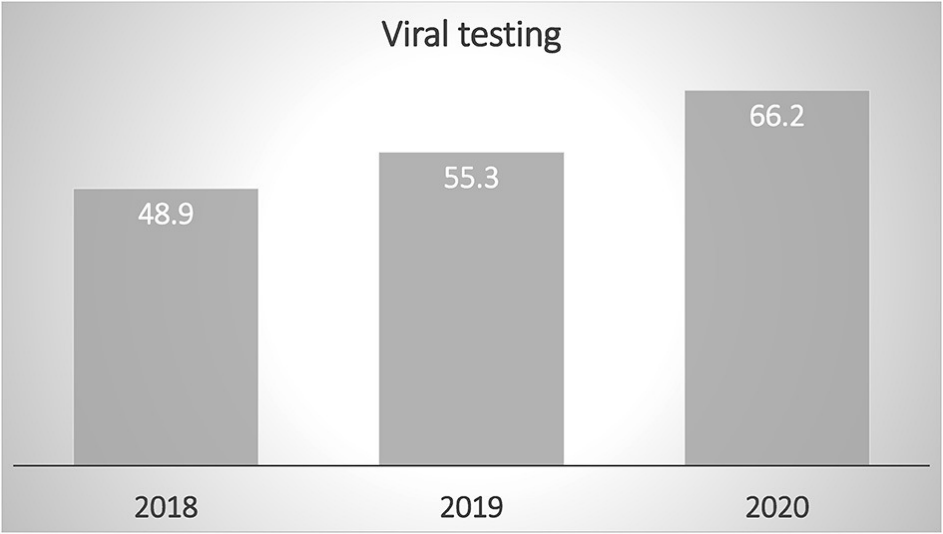
Viral testing increased during the study months of 2020, reflecting increased measures to detect viral illnesses in early phase during the early phase of the coronavirus disease pandemic.

The number of respiratory samples and identifications increased from 48.9% and 53.2% in 2018 and 2019, respectively, to 66.2% (*n* = 133) in 2020. Of the 133 patients who were tested in 2020, a virus was detected in 38.3% (*n* = 52), which was statistically higher than the positive samples obtained in 2018 and 2019 (26.4% and 21.9%, respectively; *P* < 0.01). The length of hospital stay remained almost the same throughout the study period (Figure 2).

### Parameters Predicting Decision to Admit in 2020

Multivariate binary logistic regression analysis provided insights into the predictors of admission during the 2020 study months. The patients admitted during the COVID-19 pandemic were found to be more likely to have been admitted with pneumonia compared to those with other lower respiratory illnesses (OR 2.03, *P* < 0.001). The results of the analysis showed that the children's age converged positively with their odds of admission with pneumonia. As a child's age increased by 1 year, their odds of pneumonia rose by 1.205 times (20.5% higher) than the average (*P* < 0.001, CI 1.13–1.27). The children who required supplemental oxygen upon admission were found to be significantly more likely (2.30 times more) to have pneumonia (*P* < 0.001, CI 1.7–3). However, the month of admission did not correlate significantly with the children's odds of admission with pneumonia, children's length of hospital stay, or ICU admission.

## Discussion

To our knowledge, this is the first multicenter study in the Middle East to evaluate the impact of COVID-19 precautions on the hospitalizations and outcomes of pediatric patients with lower respiratory illnesses, such as bronchiolitis, asthma, and pneumonia. This study showed that the burden of these illnesses was significantly less in the spring of 2020 than in the previous 2 years in Saudi Arabia.

In Saudi Arabia, the government announced a national lockdown on March 23, 2020, and an order for a complete curfew was placed until May 27, 2020 (6). A retrospective review was conducted using patient records from four hospitals in two major cities of Saudi Arabia that had a designated pediatric unit in a pediatric ICU. In Saudi Arabia, the pediatric population is defined as persons aged from birth until the age of 14 years (9). Similar to our findings, a previous study found a drop in admission rates due to asthma exacerbation in the Republic of Slovenia. Their study concluded a reduction of 71–78% in pediatric asthma admissions during the first 5 weeks of the implementation of state-wide COVID-19 precautions compared with the same time periods during the previous 3 years (10). Several studies have shown a decline in hospital emergency visits (11–13). Dann et al. reported a 73–88% reduction in pediatric emergency department visits during the first year of the pandemic (11). Despite the different scopes of practice between pediatric emergencies and pediatric hospitalizations, both practices can provide evidence of lower floating viral illnesses in communities. Another example came from Italy with a huge reduction in bronchiolitis throughout the bronchiolitis season, even after the ease of lockdown restriction (14).

The significant decrease in the number of hospitalizations can be explained by many reasons. We speculate that this is not solely related to hospital avoidance and late presentation. Our theory was based on a similar pattern of drops in oxygen requirements, mechanical ventilation, and the need for ICU care. The reduction of ICU care echoed another study conducted in Brazil, describing a significant decrease in pediatric ICU admissions for asthma and respiratory infections (15). Additionally, there was no increase in the length of hospital stay, which proves that the severity of hospitalized patients remained the same throughout the study period.

Our data showed a surge in viral testing, which was accompanied by early phases of the COVID-19 pandemic. This surge in testing might add more cost and burden to patients, families, and healthcare providers. As we are cruising through the second year of the COVID-19 pandemic, we need to apply clinical stewardship and practice cost-effective care. Clear guidelines should be implemented to mitigate overtesting and unnecessary investigations.

Given our study design, it is difficult to understand how children's age and oxygen requirement converge with the likelihood of being admitted due to pneumonia. This could be most likely due to the higher probability of pneumonia and lower probability of bronchiolitis in older children. As a standard of practice in many countries, oxygen requirement is a strong indicator for admission, regardless of diagnosis. However, the need for oxygen being a more pronounced factor in children with pneumonia could be due to the lower number of bronchiolitis and viral-induced asthma in our study population.

The highlight of our study was the higher probability of being admitted due to pneumonia. As this is an observational study, it is difficult to assume causality. However, this is an interesting finding. Do lockdown, social distancing, hand washing, and wearing masks shift the paradigm of infectious respiratory diseases? Will the interruption of routine vaccine schedules during the COVID-19 pandemic lead to a further rise in hospitalization secondary to pneumonia? Further research is needed to answer these questions. Additionally, the long-term impact of fewer viral illnesses and better hygiene practices on children's health needs to be fully studied. Another question arises about the preparedness of coming winter and spring seasons, as some reports speculate the rise of lower respiratory diseases upon the easing restrictions and protective measures (16). The increase in bronchiolitis cases even in unusual times has been witnessed by many around the globe (17). We believe that the impact of the COVID-19 pandemic on children's health is still beyond our comprehension.

## Conclusion

COVID-19 precaution measures, including a national lockdown, helped in lowering hospitalizations secondary to lower respiratory illnesses in Saudi Arabia. The reduction in admission numbers was accompanied by a reduction in the need for ICU care and supplemental oxygen, which disputed claims of hospital avoidance and delayed presentation. Viral testing has increased significantly during the pandemic. Despite the surge in viral testing, the likelihood of being admitted due to a diagnosis of pneumonia was higher than being admitted due to a diagnosis of COVID-19. The effectiveness of the national lockdown on COVID-19 transmission can be ensured by the significant and immediate impact of the lockdown on other lower respiratory illnesses.

## Limitations

This was a retrospective cross-sectional study based on chart review. It was subject to the quality of the documented data. The upper age limit of 14 years excluded middle and late adolescents. Despite higher viral testing, none of the study participants tested positive for COVID-19.

## Data Availability Statement

The raw data supporting the conclusions of this article will be made available by the corresponding author, without undue reservation.

## Author Contributions

NA conceived and designed the study, arranged the data collection, oversaw analysis and drafted the manuscript, and submitted the final version. AAla, AAle, and AY contributed to the design of the study, supported the data collection in their centers, provided critical revision to the manuscript, and approved the final version. YA, AAlh, and FA helped in the data collection, data recording in excel files in their centers, helped in writing the manuscript, revision, and approval of the final version. IN did the data analysis and helped in writing the methods and results sections. All authors contributed to the article and approved the submitted version.

## Funding

The authors were grateful to the Deanship of Scientific Research, King Saud University for funding through Vice Deanship of Scientific Research Chairs. The sponsor had no influence on the study design or the reporting of the results.

## Conflict of Interest

The authors declare that the research was conducted in the absence of any commercial or financial relationships that could be construed as a potential conflict of interest.

## Publisher's Note

All claims expressed in this article are solely those of the authors and do not necessarily represent those of their affiliated organizations, or those of the publisher, the editors and the reviewers. Any product that may be evaluated in this article, or claim that may be made by its manufacturer, is not guaranteed or endorsed by the publisher.
